# Complications of Cochlear Implant Surgery in Low‐ and Middle‐Income Countries: A Systematic Review

**DOI:** 10.1002/ohn.70318

**Published:** 2026-06-17

**Authors:** Akash Srinivasan, Anavi Prakash, Wing K. Chou, Kat Steiner, Kokila Lakhoo

**Affiliations:** ^1^ Medical Sciences Division University of Oxford Oxford UK; ^2^ Department of Research & Development Salisbury District Hospital Salisbury UK; ^3^ Bodleian Health Care Libraries University of Oxford Oxford UK; ^4^ Nuffield Department of Surgical Sciences University of Oxford Oxford UK

**Keywords:** cochlear implantation, complications, hearing loss, LMIC, surgery

## Abstract

**Objective:**

Hearing loss disproportionately affects low‐ and middle‐income countries (LMICs). Recent advances in cochlear implant surgery have benefitted patients globally, but the risk of complications in LMICs may be heightened due to social and structural factors. This systematic review characterises the types and rates of complications reported in LMICs, while identifying the key barriers and facilitators to safe surgery.

**Data Sources:**

Three databases (Ovid MEDLINE, Ovid Embase, and Global Index Medicus) and grey literature were searched from January 1, 2015 to May 30, 2025.

**Review Methods:**

Studies reporting quantitative data on cochlear implant surgery complications were independently reviewed by 2 authors, with conflicts resolved by a third reviewer.

**Results:**

Thirty eligible studies from seventeen countries were identified. No studies were based in low‐income countries, with 37% and 63% arising from lower‐middle‐income and upper‐middle‐income countries, respectively. Pooled average complication rates were higher in lower‐middle‐income (8.83%) than upper‐middle‐income countries (5.08%). Complication profiles also varied by income classification, with facial nerve injuries predominating in lower‐middle‐income countries compared to vertigo/dizziness and device failure in upper‐middle‐income countries. Thematic analysis identified systemic, procedural, technological, and capacity issues as barriers to safe surgery, whilst highlighting the importance of specialist expertise, perioperative planning, multidisciplinary teams, and widening access initiatives to mitigate these barriers.

**Conclusion:**

Overall complication rates of cochlear implant surgery in LMICs are comparable to high‐income countries, although data may be underreported due to limited follow‐up, and complication profiles differ by income classification. Further work is required to overcome the social and structural barriers to safe surgery.

The prevalence of hearing loss has risen significantly, with an estimated 6.1% of the global population affected by disabling hearing impairment in 2018.^1^ Untreated hearing loss has numerous negative consequences, including impaired psychosocial development in children^2^ and cognitive decline in older adults.^3^ The burden of disease is particularly amplified in low‐ and middle‐income countries (LMICs) which face the dual challenge of higher rates of hearing loss compared with high‐income countries, as well as limited treatment availability.^4^, ^5^

Cochlear implantation is a treatment option for sensorineural hearing loss, which involves the surgical implantation of a prosthesis that restores auditory function by transmitting electrical impulses directly to the cochlear nerve.^6^ The earliest prostheses consisted of a single‐channel electrode, but these have since evolved into smaller, multi‐channel devices that are more comfortable to wear.^6^ As a result of these technological advances, modern cochlear implants have demonstrated high efficacy in improving auditory outcomes, speech performance, and quality of life.^7^

There are several complications related to cochlear implant surgery, ranging from common postoperative issues such as infection and bleeding to more specific complications including cerebrospinal fluid (CSF) leak and electrode migration.^8^ As the efficacy of the procedure has improved over time, the overall complication rates have concurrently decreased due to advances in surgical technique and device biocompatibility.^9^ However, patients in LMICs may have reduced access to specialist clinicians, newer technologies, and comprehensive follow‐up care, thus elevating the risk of certain complications.^10^

The purpose of this systematic review was to identify the types and rates of clinical complications associated with cochlear implant surgery in LMICs, whilst evaluating the social and structural factors that influence these outcomes.

## Methods

A systematic review was performed in accordance with established guidance^11^ and reported as per the Preferred Reporting Items for Systematic Reviews and Meta‐Analyses (PRISMA) statement.^12^ Ethical approval was not required as individual patient‐level data were not collected. The protocol was registered with PROSPERO (CRD420251080103). World Bank definitions were used to classify countries as high‐income, upper‐middle‐income, lower‐middle income and low‐income.^13^

### Search Strategy and Study Selection

The following 3 electronic databases were searched from January 1, 2015 to May 30, 2025: Ovid MEDLINE, Ovid Embase, and Global Index Medicus. A detailed grey literature search was also performed using Google Advanced Search and LMIC health ministry websites. A search strategy, which is available in the supplementary materials (Appendix A, available online), was developed to identify cochlear implant studies conducted in LMICs as defined by the World Bank. Following the removal of duplicates, 2 reviewers independently screened the retrieved studies using Covidence (Veritas Health Innovation). Conflicts were resolved by a third reviewer after the title and abstract screening stage and after the full‐text screening stage.

Table 1 summarises the eligibility criteria for study selection.

**Table 1:** Inclusion and Exclusion Criteria

Inclusion	Exclusion
Patients in LMICs who underwent unilateral or bilateral cochlear implant surgery.	Studies including high‐income country populations unless LMIC data can be disaggregated.
Primary cochlear implant surgery (unilateral/bilateral) where complications are reported.	Non‐surgical interventions or studies where complications of primary cochlear implant surgery cannot be isolated.
Observational studies, interventional studies, case series, and relevant grey literature meeting AACODS criteria. Studies in all languages.	Case reports, reviews, editorials, and conference abstracts. Studies published before January 1, 2015. Studies with fewer than 10 patients undergoing primary cochlear implant surgery.
Studies reporting quantitative data on clinical complications of primary cochlear implant surgery for the entire cohort undertaking the procedure.	Studies selectively reporting individual complications or focusing solely on the management or salvage of specific complications.

Abbreviation: LMICs, low‐ and middle‐income countries.

### Data Extraction

Two reviewers independently extracted data from each study using a standardised template. The extracted data consisted of study, population, intervention, and health system characteristics, along with quantitative outcomes (complication rates) and qualitative outcomes (facilitators and barriers to safe surgery).

### Risk of Bias Assessment

Risk of bias was evaluated by 2 independent reviewers using the Joanna Briggs Institute (JBI) Critical Appraisal Checklists. For each included study, an appropriate JBI checklist was selected according to study design.

### Synthesis

Due to the heterogeneity of studies, a meta‐analysis was considered unsuitable. Therefore, quantitative data were summarised, and qualitative outcomes were synthesised thematically using the three‐step approach described by Thomas and Harden.^14^

## Results

The study selection process identified 30 eligible studies, as illustrated by the PRISMA flow diagram (Figure 1).^12^

**Figure 1:**
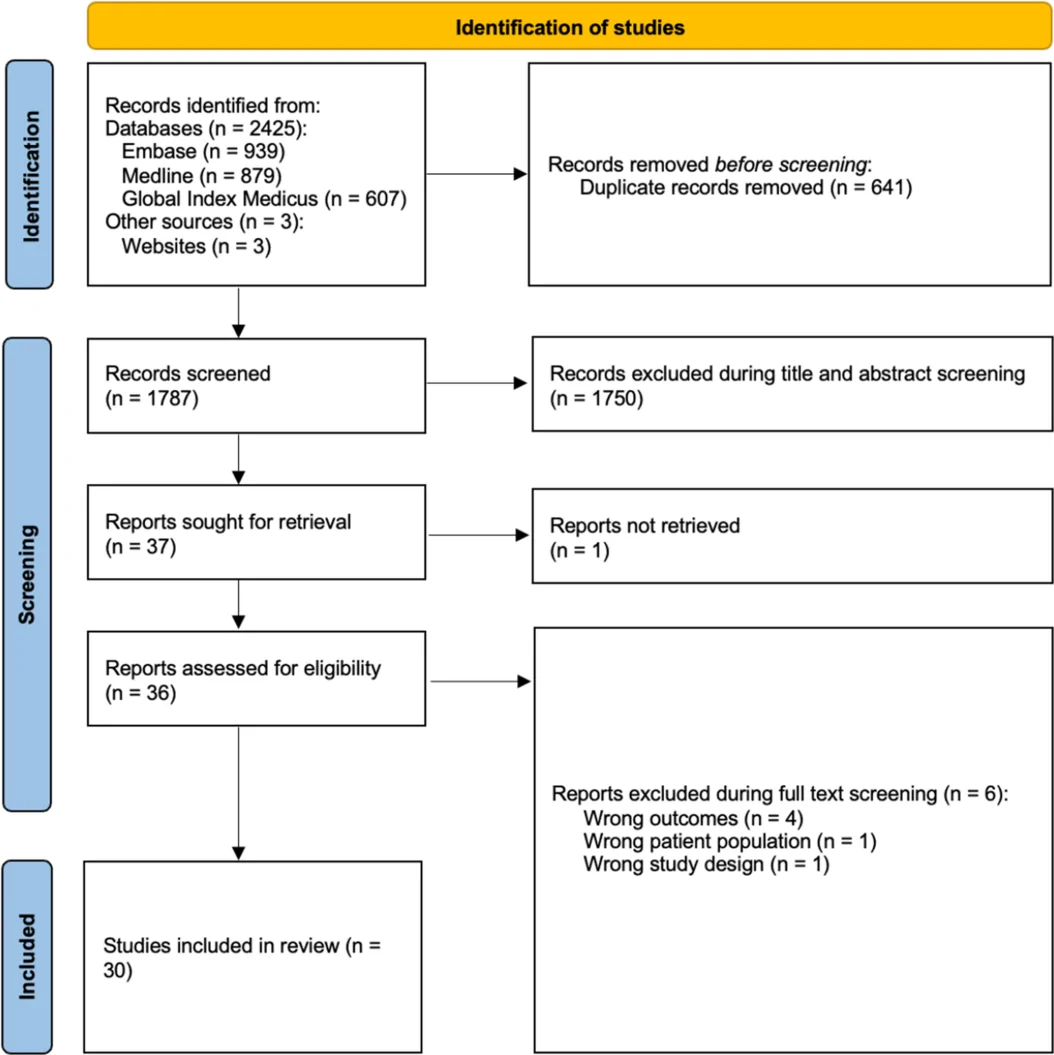
PRISMA diagram displaying the study selection process.

### Study Characteristics

The 30 included studies^15^, ^16^, ^17^, ^18^, ^19^, ^20^, ^21^, ^22^, ^23^, ^24^, ^25^, ^26^, ^27^, ^28^, ^29^, ^30^, ^31^, ^32^, ^33^, ^34^, ^35^, ^36^, ^37^, ^38^, ^39^, ^40^, ^41^, ^42^, ^43^, ^44^ were observational in design and conducted in 17 different LMICs, although 50% of the studies originated from India (n = 7), China (n = 5), and Brazil (n = 3). No studies were identified from low‐income countries at the time of publication, with lower‐middle‐income countries generating 37% of the studies (Table 2) and the remaining 63% arising from upper‐middle‐income countries (Table 3). Most studies only included pediatric patients, defined as under 16 years, but several studies either selected adults only or mixed populations. Samples sizes ranged from 10 to 4346 patients and the reported follow‐up durations varied from 5 days to 90 months. There was also significant variation between studies in terms of the surgical techniques and devices used.

**Table 2:** Summary of Studies in Lower‐Middle‐Income Countries

Study	Design	Country	Sample size	Patient characteristics	Intervention details	Follow‐up duration	Complications data
Quang (2019)^23^	Descriptive case study	Vietnam	94 patients	Pediatric patients only	Device: Cochlear Nucleus Technique: Small skin incision and minimal dissection, with electrode insertion via bony cochleostomy or round window approach.	N/A	Overall complication rate: 3.2% Most frequent complication: facial nerve palsy (2.1%)
Natarajan (2020)^25^	Retrospective observational study	India	25 patients	Adult patients only	Device: MED‐EL implants Technique: Single‐stage or two‐stage procedure, with electrode insertion via round window approach.	Mean of 8 years	No complications reported
Sarker (2020)^26^	Cross‐sectional observational study	Bangladesh	40 patients	Pediatric patients only	Device: N/A Technique: Limited cortical mastoidectomy, cochleostomy, and electrode insertion through fenestra into the scala tympani of the cochlea.	At least 4 months	Overall complication rate: 25% Most frequent complication: seroma (10%)
Ahmed (2021)^28^	Retrospective analysis	Pakistan	251 patients	Pediatric patients only	N/A	N/A	Overall complication rate: 6.4% Most frequent complications: wound infection, acute otitis media (1.2%)
Haque (2021)^27^	Case series	India	15 patients	Pediatric patients only	N/A	N/A	Overall complication rate: 26.7% Most frequent complication: post‐operative nausea and vomiting (13.3%)
Pradhananga (2022)^32^	Prospective observational study	Nepal	88 patients	Pediatric patients only	Device: Cochlear or MED‐EL implants Technique: Posterior tympanotomy.	2 weeks to 6 months	Overall complication rate: 20.5% Most frequent complication: exposed portion of facial nerve (11.4%)
Sharma (2022)^37^	Retrospective observational study	India	146 patients	Pediatric and adult patients	Device: Cochlear Nucleus CI422 Technique: Posterior tympanotomy and electrode insertion via round window approach.	At least 6 months	Overall complication rate: 12.3% Most frequent complication: magnet‐induced boil (10.3%)
Sinha (2022)^36^	Retrospective analysis	India	131 patients	Pediatric patients only	Device: N/A Technique: Mastoidectomy and posterior tympanotomy.	N/A	Overall complication rate: 3.8% Most frequent complication: transient facial nerve palsy (3.1%)
Jaiswal (2023)^38^	Retrospective chart review	India	21 patients	Adult patients only	Device: Cochlear Nucleus CI24RE (ST) Technique: Posterior tympanotomy.	Mean of 44 months	Overall complication rate: 4.8% Most frequent complication: facial nerve palsy (4.8%)
Mathews (2023)^39^	Medical record‐based analytical study	India	1250 patients	Pediatric and adult patients	Device: N/A Technique: Small post‐auricular incision, posterior tympanotomy, with electrode insertion via cochleostomy or round window approach.	1 month to 90 months	Overall complication rate: N/A Overall major complication rate: 9% Overall minor complication rate: 6% Most frequent complication: device failure (6%)
Aqib (2025)^41^	Ambispective chart review	India	50 patients	Adult patients only	Device: Cochlear or MED‐EL implants Technique: Veria technique, subtotal petrosectomy (for recurrent squamosal disease) or posterior tympanotomy (for hyperpneumatisation of the mastoid). Electrode insertion via anterior cochleostomy, anterior inferior cochleostomy, anterosuperior approach, or round window approach.	N/A	Overall complication rate: 2% Most frequent complications: dizziness, vertigo, tinnitus (2%)

**Table 3:** Summary of Studies in Upper‐Middle‐Income Countries

Study	Design	Country	Sample size	Patient characteristics	Intervention details	Follow‐up duration	Complications data
Daneshi (2015)^15^	Retrospective analysis	Iran	4346 patients	Pediatric patients only	Device: MED‐EL COMBI 40 in most cases Technique: Post‐auricular incision.	At least 1 year	Overall complication rate: 0.8% Most frequent complication: intra‐operative CSF leakage (0.4%)
Dankuc (2015)^16^	Retrospective analysis	Serbia	99 patients	Pediatric and adult patients	Device: N/A Technique: Posterior tympanotomy with a surgical navigation system.	N/A	Overall complication rate: 11.1% Most frequent complications: unsuccessful implantation, transient facial nerve paresis (4%)
Yépez‐Pabón (2015)^17^	Retrospective descriptive study	Ecuador	275 patients	Pediatric and adult patients	N/A	20 months	Overall complication rate: 12% Most frequent complication: local infection (3.3%)
Alcas (2016)^18^	Retrospective descriptive study	Peru	107 patients	Pediatric and adult patients	Device: Cochlear or MED‐EL implants Technique: N/A	At least 3 months	Overall complication rate: 18.7% Most frequent complication: tinnitus (5.6%)
Chen (2017)^19^	Retrospective observational study	China	74 patients	Pediatric patients only *Patients with large vestibular aqueduct syndrome were compared with patients with normal anatomy*	Device: MED‐EL C40+ or Cochlear Nucleus‐24 Technique: Minimally invasive mastoid‐facial recess approach with cochleostomy antero‐inferior to the round window.	At least 1 year	Overall complication rate: N/A (*0% in patients with normal anatomy)* Most frequent complication: peri‐lymphatic fluid fluctuation (54.1% of large vestibular aqueduct syndrome group)
Devesahayam (2018)^20^	Retrospective review	Malaysia	56 patients	Pediatric and adult patients	Device: Cochlear or MED‐EL implants Technique: Post‐auricular incision, posterior tympanotomy, and electrode insertion via cochleostomy or round window approach.	N/A	Overall complication rate: 3.6% Most frequent complications: electrode migration, device failure (1.8%)
Diab (2018)^21^	Retrospective observational study	Russia, Azerbaijan	847 patients	Pediatric and adult patients	Device: Oticon Medical/Neurelec, MED‐EL, Cochlear or Advanced Bionics implants Technique: Posterior tympanotomy.	2 years	Overall complication rate: N/A Overall major complication rate: 2.6% Overall minor complication rate: 1.6% Most frequent complication: technical failure of implant (2%)
Kasemsiri (2018)^22^	Retrospective and prospective observational study	Thailand	216 patients	Pediatric and adult patients	Device: Cochlear, MED‐EL, or Advanced Bionics implants Technique: N/A	Up to 5 years	Overall complication rate: 8.3% Most frequent complication: vertigo (1.5%)
Lai (2020)^24^	Retrospective analysis	China	386 patients	Pediatric patients only *Patients with inner ear malformations were compared with patients with normal anatomy*	Device: N/A Technique: Posterior tympanotomy and electrode insertion via cochleostomy or expanded round window approach.	1 month to 5 years	Overall complication rate: 0.5% Most frequent complication: CSF gusher (15.2% of inner ear malformation group)
Huang (2021)^29^	Retrospective review	China	10 patients	Adult patients only *All patients had previously received radiotherapy for nasopharyngeal carcinoma*	Device: Cochlear CI24R(ST), Cochlear CI24R(CA), Cochlear Nucleus CI422, Cochlear Nucleus CI512 Technique: Routine cochlear implantation with mastoidectomy and posterior tympanotomy (mild cases). Extended radical mastoidectomy with/without myringoplasty and simultaneous cochlear implantation (moderate cases). Subtotal temporal bone resection, external auditory canal elimination and cochlear implantation (severe cases).	Mean of 63.2 months	Overall complication rate: 10% Most frequent complication: radiation encephalopathy leading to idle implant (10%)
Piromchai (2021)^30^	Multicentre prospective cohort study	Thailand	458 patients	Pediatric and adult patients	Device: N/A Technique: Electrode insertion via cochleostomy or round window approach.	1 year	Overall complication rate: N/A Most frequent complication: device failure (4.1%)
Guo (2022)^31^	Retrospective observational study	China	38 patients	Adult patients only *Patients with tinnitus were compared with patients without tinnitus*	Device: Multi‐channel implants Technique: Post‐auricular incision, facial recess approach and electrode insertion via cochleostomy or round window approach.	4 years to 6 years	No complications reported
AlKhtoum (2022)^33^	Retrospective observational study	Jordan	840 patients	Pediatric and adult patients	Device: Cochlear Nucleus, MED‐EL, or Advanced Bionics implants Technique: Minimal post‐auricular incision and flap approach with a tight subperiosteal pocket.	At least 1 year	Overall complication rate: 3.9% Most frequent complication: hematoma (1.1%)
Castro (2022)^34^	Historical cohort study	Brazil	432 patients	Pediatric and adult patients	Device: Cochlear, MED‐EL, Advanced Bionics, or Oticon implants Technique: Mastoidectomy with a tight periosteal pocket.	At least 5 days	Overall complication rate: 15.5% Most frequent complication: dizziness (6.3%)
Ozdemir (2022)^35^	Retrospective case review study	Turkey	859 patients	Pediatric and adult patients	Device: Cochlear Nucleus, MED‐EL, Advanced Bionics, or Oticon implants Technique: Post‐auricular incision, with modifications for cases after 2018 to reduce incision size and minimise complications.	At least 1 year	Overall complication rate: 18.3% Most frequent complication: seroma‐hematoma (4%)
Orlando (2024)^40^	Retrospective study	Brazil	193 implanted ears	Pediatric and adult patients	Device: Cochlear, MED‐EL, Advanced Bionics, Oticon, or Neurelec implants Technique: Electrode insertion via cochleostomy or round window approach.	Mean of 5.4 years	Overall complication rate: N/A Overall major complication rate: 9.8% Overall minor complication rate: 29% Most frequent complication: minor vertigo (8.8%)
Li (2025)^42^	Prospective observational study	China	74 patients	Pediatric patients only	Device: N/A Technique: Minimal incision, mastoid contouring, facial recess approach, with electrode insertion via round window or expanded round window approach.	5 years to 6 years	Overall complication rate: 12.3% Most frequent complication: peri‐implant swelling or hematoma (3.5%)
Souza (2025)^43^	Retrospective observational and descriptive study	Brazil	205 patients	N/A	Device: N/A Technique: Electrode insertion via cochleostomy or round window approach.	At least 1 year	Overall complication rate: 8.7% Most frequent complication: vertigo (4.3%)
Zizlavsky (2025)^44^	Retrospective descriptive study	Indonesia	255 patients	Pediatric and adult patients	N/A	N/A	Overall complication rate: 4.3% Most frequent complication: device migration (1.6%)

Using the JBI Critical Appraisal Checklists, 4 studies were classified as having moderate risk of bias,^16^, ^23^, ^27^, ^43^ while the remaining 26 studies were at low risk of bias. The full risk of bias assessment is available in the supplementary materials (Appendix B, available online).

### Complication Rates

Twenty‐six studies reported overall complication rates, which ranged from 0% to 26.7% in lower‐middle‐income countries and 0% to 18.7% in upper‐middle‐income countries. For the studies that reported overall complication data, the mean rate per study was 10.47% and 8.53% in lower‐middle‐income and upper‐middle‐income countries, respectively. However, after adjusting for sample sizes, the pooled average complication rate was 8.83% in lower‐middle‐income studies and 5.08% in upper‐middle‐income studies.

Complication profiles also varied by income classification: facial nerve complications were the most frequent in 4 studies from lower‐middle‐income countries,^23^, ^32^, ^36^, ^38^ whereas vertigo or dizziness was the most common in four upper‐middle‐income studies^22^, ^34^, ^40^, ^43^ followed by device failure in three.^20^, ^21^, ^30^ Figure 2 summarises the 10 most frequently reported complications across the included studies. Facial nerve complications, such as palsy or transient paresis, were documented in 23 studies (77%)^15^, ^16^, ^17^, ^18^, ^22^, ^23^, ^24^, ^26^, ^27^, ^28^, ^30^, ^32^, ^33^, ^34^, ^35^, ^36^, ^37^, ^38^, ^39^, ^40^, ^42^, ^43^, ^44^ from 15 countries, including 5 studies set in India.^27^, ^36^, ^37^, ^38^, ^39^ Device failure was also reported in the majority of included studies (53%) across 14 countries.^16^, ^18^, ^20^, ^21^, ^22^, ^26^, ^28^, ^30^, ^32^, ^33^, ^34^, ^35^, ^39^, ^40^, ^43^, ^44^ Other notable complications included vertigo/dizziness, wound and ear infections, and electrode position changes such as migration or extrusion.

**Figure 2:**
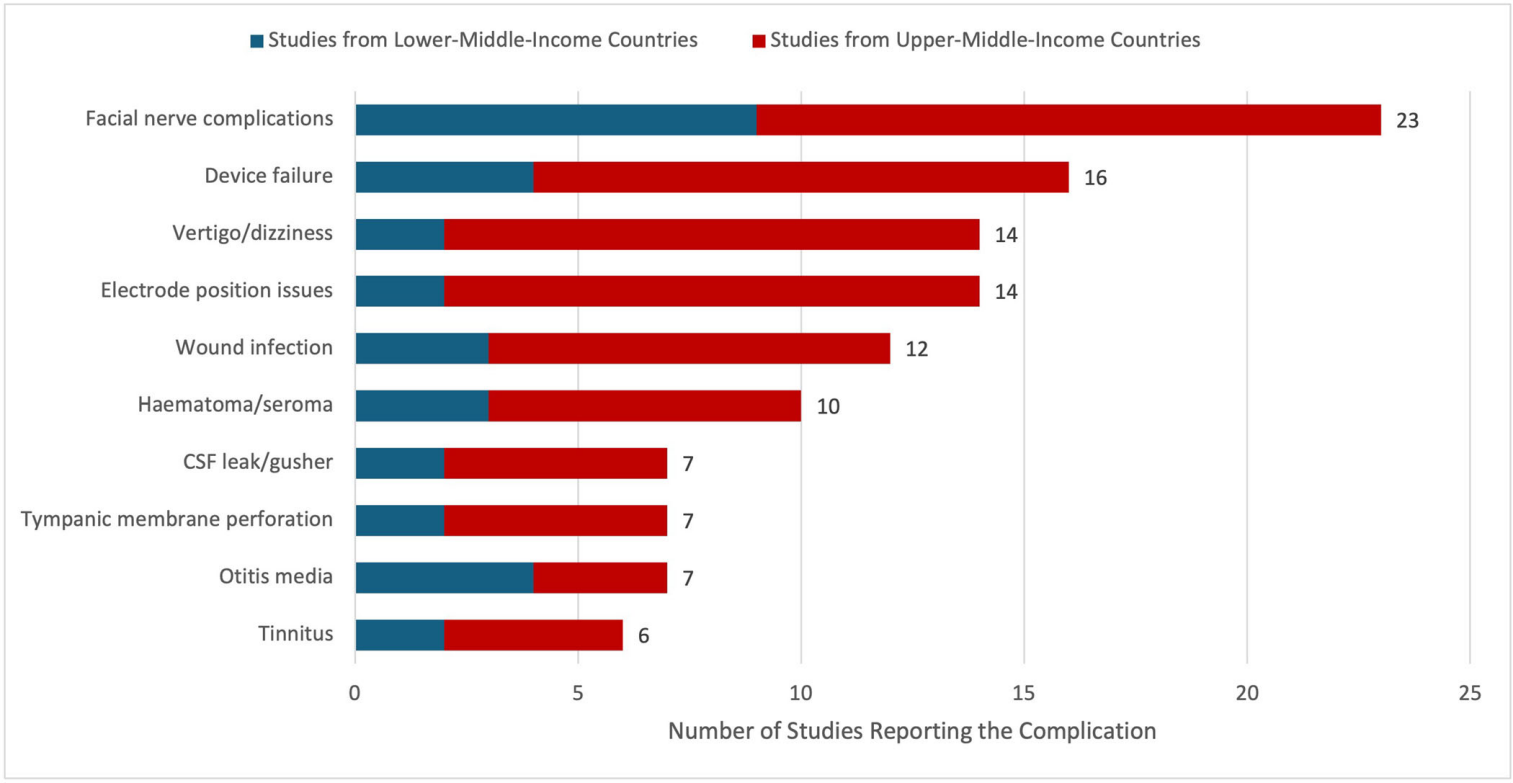
Ten most frequently reported complications.

### Barriers and Facilitators to Safe Surgery

Studies were analysed to identify social and structural factors influencing complication outcomes. Thematic analysis was performed to identify barriers and facilitators to safe cochlear implant surgery in LMICs, with the analytical themes outlined in Figure 3. The full process of line‐by‐line coding and synthesis of descriptive and analytical themes is described in the supplementary materials (Appendix C, available online).

**Figure 3:**
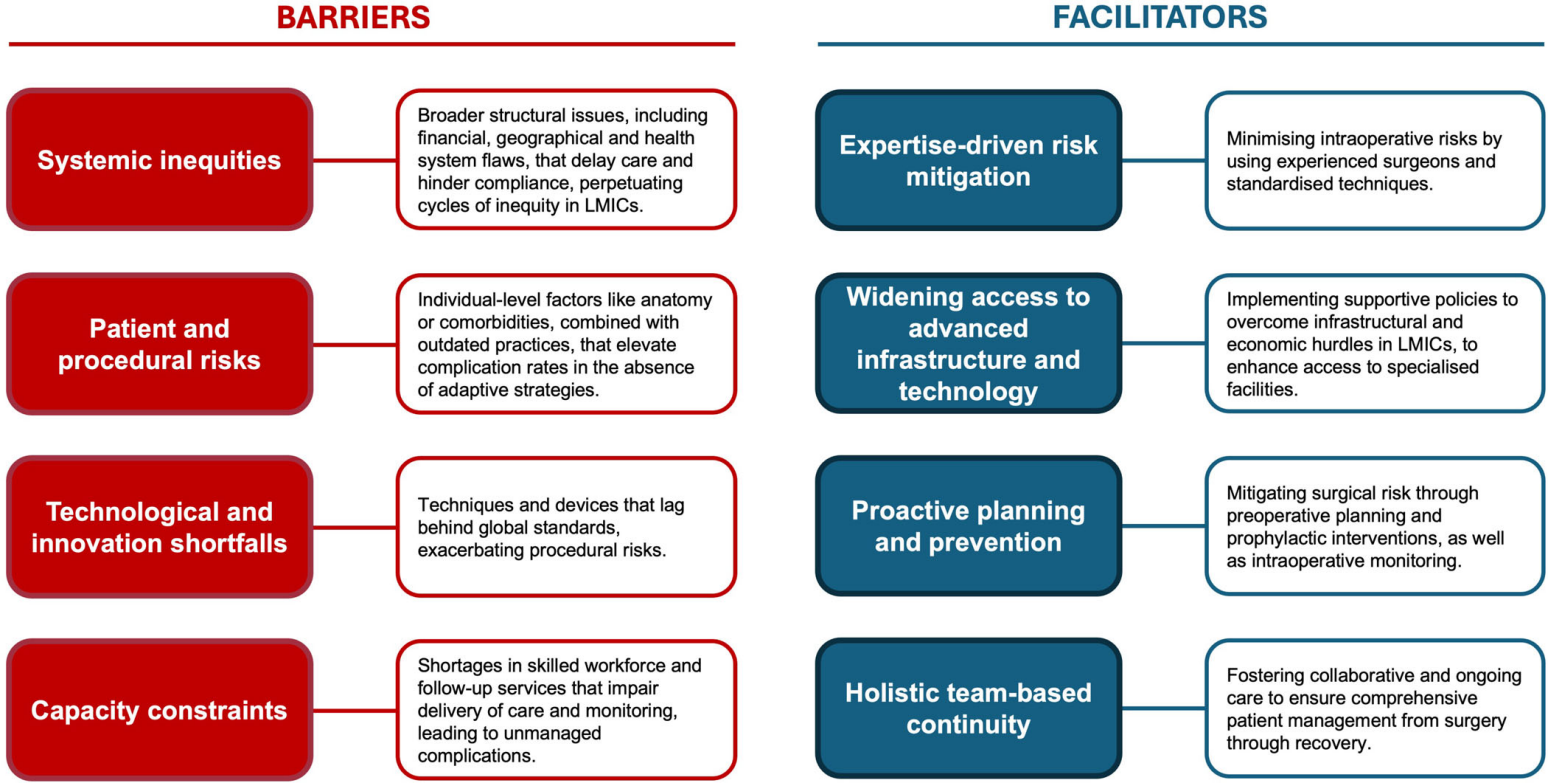
Analytical themes for barriers and facilitators to safe cochlear implant surgery in LMICs. LMICs, low‐ and middle‐income countries.

Four analytical themes were synthesised in terms of barriers to safe surgery: systemic inequities, patient and procedural risks, technological and innovation shortfalls, and capacity constraints. Systemic inequities arise from several factors, including limitations in public health infrastructure,^34^ the costs associated with surgery and follow‐up,^44^ and geographical barriers preventing specialists from accessing rural locations^44^ as well as patients from complying with follow‐up.^34^ Patient and procedural risks were highlighted by multiple studies where anatomical abnormalities^24^ or comorbid ear infections^25^, ^40^ increased the complexity of surgery, especially when managed by less experienced surgeons.^19^ Technological shortfalls were exemplified in the study by Sarker et al,^26^ where 3 cases of transient facial nerve paresis were caused by overheating during drilling. Moreover, fewer device‐related complications are seen with new generation implants,^15^ but high‐income countries typically benefit from these innovations ahead of LMICs. Lastly, capacity constraints are seen to consistently affect LMICs, where specialised tertiary centres may have the ability to deliver high quality care but struggle to provide adequate coverage for the overall population.^44^

Conversely, four analytical themes for facilitators to safe surgery were also identified: expertise‐driven risk mitigation, widening access to advanced infrastructure and technology, proactive planning and prevention, and holistic team‐based continuity. The use of expertise to minimise risk, with experienced surgeons either performing the operations^32^, ^33^ or supervising trainees,^21^, ^39^ was frequently credited with lowering complication rates. Although cost is a major barrier for many potential patients, Sinha et al^36^ and Alcas et al^18^ demonstrate positive examples of public funding schemes mitigating this issue. Private companies such as Cochlear and MED‐EL have also helped improve accessibility by training clinicians in Nepal.^32^ Proactive planning and prevention includes the use of preoperative imaging such as MRI,^24^, ^37^ prophylactic vaccinations,^15^, ^16^ antibiotics,^33^, ^35^ and intraoperative facial nerve monitoring^20^, ^35^ to help anticipate and manage surgical challenges. Finally, involving the multidisciplinary team to comprehensively evaluate patients preoperatively whilst delivering holistic postoperative care and follow‐up helps to reduce avoidable complications.^18^, ^28^

## Discussion

The use of cochlear implantation in high‐income countries has expanded in recent years,^45^, ^46^ yet the proportion of potential users across the world who have an implant is estimated to be as low as 1%.^47^ Uptake in LMICs is primarily inhibited by logistical barriers including the cost of devices and surgery,^47^ but addressing technical issues such as complications is also vital to improving patient confidence in the intervention.

This systematic review synthesises data from 30 studies, published between 2015 and 2025, reporting the complications of cochlear implant surgery in LMICs. These studies demonstrate substantial heterogeneity in patient populations, reporting methods, definitions of complications, and follow‐up durations. Consequently, a wide range of overall complication rates were reported, regardless of income classification, although the average rate was higher across lower‐middle‐income studies than higher‐middle‐income studies. There was also a notable difference between these countries in the types of complications reported: immediate facial nerve injuries predominated in lower‐middle‐income studies which may result from less routine use of facial nerve monitoring in resource‐constrained settings. Meanwhile, longer‐term issues such as device failure and electrode migration were more prominent in higher‐middle‐income studies. This likely reflects the greater access to extended follow‐up in wealthier nations.

The outcomes described in the literature for high‐income countries are comparable to the LMIC data in this review, with a retrospective study of 434 cases in France reporting an overall complication rate of 9.9%.^48^ However, it should be noted that the majority of these complications were delayed, occurring after an average delay of 2.2 years, which indicates that complications may be underreported in LMIC studies with shorter follow‐up durations. Similarly, Deep et al^49^ demonstrated a complication rate of 14% among a cohort of infants, and 53% of these complications were defined as “late” (presenting over 30 days postoperatively). Notably, there were no instances of device‐related complications in this study,^49^ in contrast to the prominent issue of device failure in LMICs. This may reflect better access to the newest technologies in high‐income countries.

Surgical complications only represent one facet of successful cochlear implantation, as there are other nonoperative factors that influence outcomes. Inadequate audiological follow‐up and aural rehabilitation are well‐documented issues in LMICs^50^ that can limit the benefits of surgery.^51^, ^52^ Interestingly, patients in high‐income countries are vulnerable to these challenges as well,^53^ particularly in rural areas where patients often have to travel long distances to receive hearing healthcare.^54^, ^55^ A range of solutions to mitigate the urban‐rural disparity have been identified including satellite clinics, telemedicine, and remote cochlear implant programming,^52^, ^54^ which may be translatable to LMICs. Long‐term outcomes are also worsened by device non‐use, which may in itself be an indicator of unsuccessful surgery if it stems from patients experiencing a complication or low auditory performance.^56^ However, none of the included studies reported daily hours of device use, thus highlighting the need for such data to be collected in a standardised manner during future research.

The absence of complication data from low‐income countries in the literature is striking and likely reflects a severe lack of cochlear implant uptake in these settings. Stevens et al^5^ suggested that hearing aid coverage in developing countries is small to negligible due to cost, and thus cochlear implant provision is presumably even lower. A 2024 editorial described the extensive hurdles faced when developing a cochlear implant programme in Malawi, including the extremely low numbers of ENT surgeons, audiologists and speech therapists, inability to attend rehabilitation, and disability‐associated stigma.^57^ The cost of battery replacement was another financial burden for patients in this setting, whilst the use of rechargeable devices was challenged by inconsistent power supplies and required the development of solar‐powered charging stations.^58^ Conflict and political instability are also often present in low‐income countries, which pose further obstacles to the delivery of hearing healthcare; Alier et al^59^ highlighted that even the use of low‐cost hearing devices in South Sudan was affected by device maintenance, repair infrastructure, and battery access challenges. Until the barriers to implementing cochlear implant programs more widely in low‐income countries are addressed, it is unlikely that any robust studies reporting complication data will emerge.

Management of complications was inconsistently reported by the included studies. Ozdemir et al^35^ described a range of management approaches including surgical drainage and pressure dressing application for hematomas, myringotomy and intravenous antibiotics for mastoiditis, as well as explantation and re‐implantation surgery for all device‐related complications. Daneshi et al^15^ reported that their cases of complete flap failure were managed using rotational musculocutaneous or free forearm flaps, whilst magnet sores were addressed by using weaker magnets. However, the majority of included studies did not provide details regarding complication management, hence precluding comparison.

This systematic review has limitations: firstly, the manual process of searching the literature and screening the reports may have led to mistakenly excluded studies. The heterogenous nature of the included studies precluded meta‐analysis and limited any robust comparisons of quantitative outcomes. Although there were benefits to excluding small studies with fewer than 10 patients, this may have led to insights from underrepresented low‐income regions being omitted. Finally, half of the included studies in this review were based in India, China, and Brazil. Whilst these countries are classified as LMICs based on gross national income per capita and display substantial wealth inequality,^13^, ^60^ all 3 countries are amongst the ten largest economies in the world.^61^ Therefore, the tertiary referral centres that perform the procedure in these countries are often advanced institutions with excellent facilities that are not generalisable to other LMICs. They face challenges in providing equitable access to healthcare,^62^ but this does not affect the technical process of performing safe cochlear implant surgery.

In conclusion, the increasing uptake of cochlear implant surgery in LMICs is a positive development, but complication rates remain substantial due to social and structural barriers. Moreover, these reported rates may be underestimated given the limited follow‐up capacity in LMICs compared to high‐income countries. Future research should adopt a more standardised and comprehensive approach to data collection, encompassing complication management, device usage, and audiological follow‐up. Further work is required to develop targeted solutions to strengthen healthcare systems, enhance surgical safety, and extend cochlear implantation programs into low‐income countries to overcome the global burden of untreated hearing loss.

## Author Contributions

**Akash Srinivasan**, designed the work, acquired and analysed data, drafted the manuscript, revised and approved the manuscript, agrees to be accountable for all aspects of the work; **Anavi Prakash**, designed the work, acquired and analysed data, revised and approved the manuscript, agrees to be accountable for all aspects of the work; **Wing K. Chou**, designed the work, acquired and analysed data, revised and approved the manuscript, agrees to be accountable for all aspects of the work; **Kat Steiner**, acquired and analysed data, agrees to be accountable for all aspects of the work; **Kokila Lakhoo**, designed the work, revised and approved the manuscript, agrees to be accountable for all aspects of the work.

## Disclosures

### Competing interests

None.

### Funding source

None.

## Supporting information


**Appendix A.** Full search strategy and results of the literature search;
**Appendix B.** Risk of bias assessment;
**Appendix C.** Thematic analysis process.

## Data Availability

Data sharing is not applicable to this article as no new data were created or analysed in this study.
