# Supplementary material for: Complications of Cochlear Implant Surgery in Low‐ and Middle‐Income Countries: A Systematic Review
Source: Otolaryngol Head Neck Surg. 2026 Jun 17;175(4):789–801. doi: 10.1002/ohn.70318 (PMC13655758; doi:10.1002/ohn.70318)
Supplement: Supplementary file 1 [file OHN-175-789-s001.docx]

**Complications of Cochlear Implant Surgery in Low- and Middle-Income Countries: A Systematic Review**

**Appendix A:** **Full search strategy and results of the literature search.**

**Ovid MEDLINE**

| **Lines** | **Search term** | **Search results** |
| --- | --- | --- |
| 1 | Cochlear Implants/ | 13880 |
| 2 | (cochlea* prosthe* or cochlea* implant*).tw,kf. | 20366 |
| 3 | 1 or 2 | 21822 |
| 4 | (afghan* or africa* or albania* or algeria* or angola* or antigua* or barbuda* or argentin* or armenia* or aruba* or azerbaijan* or bahrain* or bangladesh* or bengal* or bangal* or barbados* or barbadian* or bajan or bajans or belarus* or belorus* or byelarus* or byelorus* or belize* or benin* or dahomey or bhutan* or bolivia* or bosnia* or herzegovin* or botswan* or batswan* or bechuanaland* or brazil* or brasil* or bulgaria* or burkina* or burkinese* or upper volta* or burundi* or urundi* or cabo verde* or cape verde* or cambodia* or kampuchea* or khmer* or cameroon* or cameroun* or ubangi shari* or chad* or chile* or china* or chinese or colombia* or comoro* or comore* or comorian* or mayotte* or congo* or zaire* or costa rica* or "cote d'ivoir*" or "cote d' ivoir*" or cote divoir* or cote d ivoir* or ivory coast* or ivorian* or croatia* or cuba or cuban or cubans or "cuba's" or cyprus* or cypriot* or czech* or djibouti* or french somaliland* or dominica* or ecuador* or egypt* or united arab republic* or el salvador* or salvadoran* or (guinea* not guinea pig*) or equatoguinea* or eritrea* or estonia* or eswatini* or swaziland* or swazi* or swati* or ethiopia* or fiji* or gabon* or gabonese* or gabonaise* or gambia* or ((georgia or georgian or georgians) not (atlanta or california or florida)) or ghana* or gibraltar* or greece* or greek* or grecian* or grenada* or grenadian* or guam* or guatemala* or guyana* or guiana* or guyanese* or haiti* or hispaniola* or hondura* or hungary* or hungarian* or india* or indonesia* or iran* or iraq* or isle of man* or jamaica* or jordan* or kazakh* or kenya* or karabati* or korea* or kosovo* or kosova* or kyrgyz* or kirgiz* or kirghiz* or laos or lao or laotian* or latvia* or lebanon* or lebanese* or lesotho* or lesothan* or lesothonian* or basutoland* or mosotho* or basotho* or liberia* or libya* or jamahiriya* or lithuania* or macedonia* or madagasca* or malagasy* or malawi* or nyasaland* or malaysia* or malay* federation or maldives* or maldivian* or indian ocean or mali or malian* or "mali's" or malta or maltese* or "malta's" or micronesia* or marshallese* or kiribati* or marshall island* or nauru or nauran or nauruans or "naurian's" or mariana or marianas or palau or paluan* or tuvalu* or mauritania* or mauritan* or mauritius* or mexico* or mexican* or moldova* or moldovia* or mongol* or montenegr* or morocco* or moroccan* or ifni or mozambique* or mozambican* or myanmar* or burma* or burmese or namibia* or nepal* or new caledonia* or netherlands antill* or nicaragua* or niger* or oman or omani or omanis or "oman's" or pakistan* or palestin* or gaza* or west bank* or panama* or paraguay* or peru or peruvian* or "peru's" or philippine* or philipine* or phillipine* or phillippine* or filipino* or filipina* or poland* or polish or pole or poles or portugal* or portuguese or puerto ric* or romania* or russia* or ussr* or soviet* or rwanda* or rwandese or ruanda* or ruandese or samoa* or navigator island* or pacific island* or polynesia* or sao tome* or santomean* or saudi arabia* or saudi or saudis or senegal* or serbia* or seychell* or sierra leone* or slovak* or sloven* or melanesia* or solomon island* or norfolk island* or somali* or sri lanka* or ceylon* or saint kitts or st kitts or kittian* or nevisian* or saint lucia* or st lucia* or saint vincent* or st vincent* or vincentian* or grenadine* or sudan* or surinam* or syria* or tajik* or tadjik* or tadzhik* or tanzania* or tanganyika* or thai* or timor leste* or east timor* or timorese* or togo or togoles* or "togo's" or tonga* or trinidad* or tobago* or tunisia* or turkiy* or turkey* or turk or turks or turkish or turkmen* or uganda* or ukrain* or uruguay* or uzbek* or vanuatu* or new hebrides* or venezuela* or vietnam* or viet nam* or yemen* or yugoslav* or zambia* or zimbabwe* or rhodesia* or arab* countr* or middle east* or global south or sahara* or subsahara* or magreb* or maghrib* or west indies* or caribbean* or central america* or latin america* or south america* or asia central or central asia* or asia northern or north asia* or northern asia* or asia southeastern or southeastern asia* or south eastern asia* or southeast asia* or south east asia* or asia western or west asia* or western asia* or europe eastern or east europe* or eastern europe* or developing countr* or developing nation* or developing population* or developing world or less developed countr* or less developed nation* or less developed world or lesser developed countr* or lesser developed nation* or lesser developed world or under developed countr* or under developed nation* or under developed world or underdeveloped countr* or underdeveloped nation* or underdeveloped world or middle income countr* or middle income nation* or middle income population* or low income countr* or low income nation* or low income population* or lower income countr* or lower income nation* or lower income population* or underserved countr* or underserved nation* or underserved population* or under served population* or under served nation* or under served population* or deprived countr* or deprived population* or high burden countr* or high burden nation* or countdown countr* or countdown nation* or poor countr* or poor nation* or poor population* or poor world or poorer countr* or poorer nation* or poorer population* or poorer world or developing econom* or less developed econom* or underdeveloped econom* or under developed econom* or middle income econom* or low income econom* or lower income econom* or low gdp or low gnp or low gross domestic or low gross national or lower gdp or lower gnp or lower gross domestic or lower gross national or lmic or lmics or third world or lami countr* or transitional countr* or emerging econom* or emerging nation*).ti,ab,hw,kf. | 3456869 |
| 5 | 3 and 4 | 1317 |
| 6 | limit 5 to yr="2015 -Current" | 879 |

**Ovid Embase**

| **Lines** | **Search term** | **Search results** |
| --- | --- | --- |
| 1 | exp cochlea prosthesis/ | 21670 |
| 2 | (cochlea* prosthe* or cochlea* implant*).tw,kf. | 23707 |
| 3 | 1 or 2 | 26886 |
| 4 | (afghan* or africa* or albania* or algeria* or angola* or antigua* or barbuda* or argentin* or armenia* or aruba* or azerbaijan* or bahrain* or bangladesh* or bengal* or bangal* or barbados* or barbadian* or bajan or bajans or belarus* or belorus* or byelarus* or byelorus* or belize* or benin* or dahomey or bhutan* or bolivia* or bosnia* or herzegovin* or botswan* or batswan* or bechuanaland* or brazil* or brasil* or bulgaria* or burkina* or burkinese* or upper volta* or burundi* or urundi* or cabo verde* or cape verde* or cambodia* or kampuchea* or khmer* or cameroon* or cameroun* or ubangi shari* or chad* or chile* or china* or chinese or colombia* or comoro* or comore* or comorian* or mayotte* or congo* or zaire* or costa rica* or "cote d'ivoir*" or "cote d' ivoir*" or cote divoir* or cote d ivoir* or ivory coast* or ivorian* or croatia* or cuba or cuban or cubans or "cuba's" or cyprus* or cypriot* or czech* or djibouti* or french somaliland* or dominica* or ecuador* or egypt* or united arab republic* or el salvador* or salvadoran* or (guinea* not guinea pig*) or equatoguinea* or eritrea* or estonia* or eswatini* or swaziland* or swazi* or swati* or ethiopia* or fiji* or gabon* or gabonese* or gabonaise* or gambia* or ((georgia or georgian or georgians) not (atlanta or california or florida)) or ghana* or gibraltar* or greece* or greek* or grecian* or grenada* or grenadian* or guam* or guatemala* or guyana* or guiana* or guyanese* or haiti* or hispaniola* or hondura* or hungary* or hungarian* or india* or indonesia* or iran* or iraq* or isle of man* or jamaica* or jordan* or kazakh* or kenya* or karabati* or korea* or kosovo* or kosova* or kyrgyz* or kirgiz* or kirghiz* or laos or lao or laotian* or latvia* or lebanon* or lebanese* or lesotho* or lesothan* or lesothonian* or basutoland* or mosotho* or basotho* or liberia* or libya* or jamahiriya* or lithuania* or macedonia* or madagasca* or malagasy* or malawi* or nyasaland* or malaysia* or malay* federation or maldives* or maldivian* or indian ocean or mali or malian* or "mali's" or malta or maltese* or "malta's" or micronesia* or marshallese* or kiribati* or marshall island* or nauru or nauran or nauruans or "naurian's" or mariana or marianas or palau or paluan* or tuvalu* or mauritania* or mauritan* or mauritius* or mexico* or mexican* or moldova* or moldovia* or mongol* or montenegr* or morocco* or moroccan* or ifni or mozambique* or mozambican* or myanmar* or burma* or burmese or namibia* or nepal* or new caledonia* or netherlands antill* or nicaragua* or niger* or oman or omani or omanis or "oman's" or pakistan* or palestin* or gaza* or west bank* or panama* or paraguay* or peru or peruvian* or "peru's" or philippine* or philipine* or phillipine* or phillippine* or filipino* or filipina* or poland* or polish or pole or poles or portugal* or portuguese or puerto ric* or romania* or russia* or ussr* or soviet* or rwanda* or rwandese or ruanda* or ruandese or samoa* or navigator island* or pacific island* or polynesia* or sao tome* or santomean* or saudi arabia* or saudi or saudis or senegal* or serbia* or seychell* or sierra leone* or slovak* or sloven* or melanesia* or solomon island* or norfolk island* or somali* or sri lanka* or ceylon* or saint kitts or st kitts or kittian* or nevisian* or saint lucia* or st lucia* or saint vincent* or st vincent* or vincentian* or grenadine* or sudan* or surinam* or syria* or tajik* or tadjik* or tadzhik* or tanzania* or tanganyika* or thai* or timor leste* or east timor* or timorese* or togo or togoles* or "togo's" or tonga* or trinidad* or tobago* or tunisia* or turkiy* or turkey* or turk or turks or turkish or turkmen* or uganda* or ukrain* or uruguay* or uzbek* or vanuatu* or new hebrides* or venezuela* or vietnam* or viet nam* or yemen* or yugoslav* or zambia* or zimbabwe* or rhodesia* or arab* countr* or middle east* or global south or sahara* or subsahara* or magreb* or maghrib* or west indies* or caribbean* or central america* or latin america* or south america* or asia central or central asia* or asia northern or north asia* or northern asia* or asia southeastern or southeastern asia* or south eastern asia* or southeast asia* or south east asia* or asia western or west asia* or western asia* or europe eastern or east europe* or eastern europe* or developing countr* or developing nation* or developing population* or developing world or less developed countr* or less developed nation* or less developed world or lesser developed countr* or lesser developed nation* or lesser developed world or under developed countr* or under developed nation* or under developed world or underdeveloped countr* or underdeveloped nation* or underdeveloped world or middle income countr* or middle income nation* or middle income population* or low income countr* or low income nation* or low income population* or lower income countr* or lower income nation* or lower income population* or underserved countr* or underserved nation* or underserved population* or under served population* or under served nation* or under served population* or deprived countr* or deprived population* or high burden countr* or high burden nation* or countdown countr* or countdown nation* or poor countr* or poor nation* or poor population* or poor world or poorer countr* or poorer nation* or poorer population* or poorer world or developing econom* or less developed econom* or underdeveloped econom* or under developed econom* or middle income econom* or low income econom* or lower income econom* or low gdp or low gnp or low gross domestic or low gross national or lower gdp or lower gnp or lower gross domestic or lower gross national or lmic or lmics or third world or lami countr* or transitional countr* or emerging econom* or emerging nation*).ti,ab,hw,kf. | 4216077 |
| 5 | 3 and 4 | 1573 |
| 6 | limit 5 to yr="2015 -Current" | 939 |

**Global Index Medicus**

| **Lines** | **Search term** | **Search results** |
| --- | --- | --- |
| Title, abstract, subject | ("cochlear implant" OR "cochlear implants" OR "cochlear implantation" OR "cochlea implant" OR "cochlea implants" OR "cochlea implantation") OR ("cochlear prosthesis" OR "cochlear prostheses" OR "cochlear prosthetic" OR "cochlear prosthetics" OR "cochlea prosthesis" OR "cochlea prostheses" OR "cochlea prosthetic" OR "cochlea prosthetics") | 1355 |
| Limit to | Publication 2015-2025 | 607 |

**Google Advanced Search**

| **Search term** | ("cochlear implant" OR "cochlear prosthesis") AND complications AND ("developing country" OR LMIC OR "low-income" OR "middle-income") filetype:pdf |
| --- | --- |

**Appendix B:** **Risk of bias assessment.**

| **Study** | **Q1: Clear inclusion criteria** | **Q2: Consecutive/complete inclusion** | **Q3: Description of participants & setting** | **Q4: Standard, reliable outcome measurement** | **Q5: Clear reporting of complications** | **Q6: Adequate follow-up period** | **Q7: Strategies for managing confounding** | **Q8: Appropriate statistical analysis** | **Q9: Overall risk of bias** |
| --- | --- | --- | --- | --- | --- | --- | --- | --- | --- |
| Ahmed (2021) | Yes | Yes | Yes | Yes | Yes | Yes | N/A | Yes | Low |
| Alcas (2016) | Yes | Yes | Yes | Yes | Yes | Unclear | N/A | Yes | Low |
| AlKhtoum (2022) | Yes | Yes | Yes | Yes | Yes | Unclear | N/A | Yes | Low |
| Aqib (2025) | Yes | Yes | Yes | Yes | Yes | Unclear | N/A | Yes | Low |
| Castro (2022) | Yes | Yes | Yes | Yes | Yes | Unclear | N/A | Yes | Low |
| Chen (2017) | Yes | Unclear | Yes | Yes | Yes | Yes | N/A | Yes | Low |
| Daneshi (2015) | Yes | Yes | Yes | Unclear | Yes | Yes | N/A | Yes | Low |
| Dankuc (2015) | Yes | Yes | Yes | Unclear | Yes | Unclear | N/A | Yes | Moderate |
| Devesahayam (2018) | Yes | Yes | Yes | Yes | Yes | Yes | N/A | Yes | Low |
| Diab (2018) | Yes | Yes | Yes | Yes | Yes | Yes | N/A | Yes | Low |
| Guo (2022) | Yes | Yes | Yes | Yes | Yes | Yes | N/A | Yes | Low |
| Haque (2021) | Yes | Yes | Yes | Yes | Unclear | Unclear | N/A | Yes | Moderate |
| Huang (2021) | Yes | Yes | Yes | Yes | Yes | Yes | N/A | Yes | Low |
| Jaiswal (2023) | Yes | Yes | Yes | Yes | Yes | Yes | N/A | Yes | Low |
| Kasemsiri (2018) | Yes | Yes | Yes | Yes | Unclear | Yes | N/A | Yes | Low |
| Lai (2020) | Yes | Yes | Yes | Yes | Yes | Yes | N/A | Yes | Low |
| Li (2025) | Yes | Yes | Yes | Yes | Yes | Yes | N/A | Yes | Low |
| Mathews (2023) | Yes | Yes | Yes | Yes | Yes | Yes | N/A | Yes | Low |
| Natarajan (2020) | Yes | Yes | Yes | Yes | Yes | Yes | N/A | Yes | Low |
| Orlando (2024) | Yes | Yes | Yes | Yes | Yes | Yes | N/A | Yes | Low |
| Ozdemir (2022) | Yes | Yes | Yes | Yes | Yes | Yes | N/A | Yes | Low |
| Piromchai (2021) | Yes | Yes | Yes | Yes | Yes | Yes | N/A | Yes | Low |
| Pradhananga (2022) | Yes | Yes | Yes | Yes | Yes | Unclear | N/A | Yes | Low |
| Quang (2019) | Yes | Unclear | Yes | Yes | Yes | Unclear | N/A | Yes | Moderate |
| Sharma (2022) | Yes | Yes | Yes | Yes | Yes | Unclear | N/A | Yes | Low |
| Sinha (2022) | Yes | Yes | Yes | Yes | Yes | Unclear | N/A | Yes | Low |
| Yépez‑Pabón (2015) | Yes | Yes | Yes | Yes | Yes | Yes | N/A | Yes | Low |
| Sarkar (2020) | Yes | Yes | Yes | Yes | Yes | Unclear | N/A | Yes | Low |
| Souza (2025) | Yes | Unclear | Yes | Yes | Yes | Unclear | N/A | Yes | Moderate |
| Zizlavsky (2025) | Yes | Yes | Yes | Yes | Yes | Unclear | N/A | Yes | Low |

**Appendix C:** **Thematic analysis process.**

1. **Thematic analysis of barriers to safe surgery**
   a. *Line-by-line coding*

| **Study** | **Codes** |
| --- | --- |
| Daneshi (2015) | outdated technology |
| Dankuc (2015) | N/A |
| Yepez-Pabon (2015) | poor postoperative management, inadequate parental education, non-compliance with care instructions, inadequate patient education, poor infection control |
| Alcas (2016) | geographic barriers to follow-up |
| Chen (2017) | anatomical abnormalities increasing risk, inadequate surgeon experience with anomalies |
| Devesahayam (2018) | N/A |
| Diab (2018) | N/A |
| Kasemsiri (2018) | pre-surgery communication limitations, limited access to hearing aids |
| Quang (2019) | outdated surgical techniques |
| Lai (2020) | anatomical abnormalities increasing risk |
| Natarajan (2020) | increased risk in infected ears |
| Haque (2021) | N/A |
| Ahmed (2021) | N/A |
| Huang (2021) | post-radiation complications |
| Piromchai (2021) | loss to follow-up |
| Guo (2022) | N/A |
| Pradhananga (2022) | late detection, delayed intervention |
| AlKhtoum (2022) | risk of implant trauma in children |
| Castro (2022) | age as risk factor, loss to follow-up, affordability of transportation, missed therapy appointments, imperfect public health system |
| Ozdemir (2022) | limited surgical capacity |
| Sinha (2022) | delayed presentation |
| Sharma (2022) | geographic barriers to follow-up, low socioeconomic status |
| Jaiswal (2023) | delayed intervention |
| Mathews (2023) | lack of surgical experience, anatomical abnormalities increasing risk, detection limitations, device failures, limited devices in LMICs, limited follow-up |
| Orlando (2024) | prior otitis media increasing risk, device-anatomy mismatch |
| Aqib (2025) | delayed presentation, inaccessibility of tertiary centres, limited follow-up |
| Li (2025) | longer surgical time, increased anaesthetic time, age as risk factor |
| Sarkar (2020) | technological limitations |
| Souza (2025) | surgical technique as risk factor |
| Zizlavsky (2025) | lack of government support, financial burden, unsafe cost-cutting, geographical barriers to follow-up, lack of specialist surgeons, lack of MDT in remote areas, poor internet connectivity, barriers to remote rehabilitation |

b. *Descriptive Themes*

| **Descriptive Theme** | **Description** | **Example Codes** |
| --- | --- | --- |
| 1. Socioeconomic and geographic access issues | Financial and locational barriers to timely intervention and compliance with follow-up. | - Financial burden. - Geographic barriers to follow-up. - Imperfect public health system. |
| 2. Delayed detection, presentation and intervention | Late assessment and diagnosis leading to increased surgical complexity. | - Delayed presentation. - Late detection. - Delayed intervention. |
| 3. Patient-related risks | Anatomical factors, comorbidities or demographics affecting the surgical risk profile. | - Anatomical abnormalities increasing risk. - Age as risk factor. - Prior otitis media increasing risk. |
| 4. Technological limitations | Outdated devices or inability to tailor implants to the patient resulting in complications. | - Technological limitations. - Outdated technology. - Device-anatomy mismatch. |
| 5. Workforce and experience gaps | Shortages in personnel and capacity. | - Lack of surgical experience. - Limited surgical capacity. - Lack of specialist surgeons. |
| 6. Follow-up and compliance challenges | Inadequate engagement with postoperative follow-up. | - Loss to follow-up. - Inadequate patient education. - Non-compliance with care instructions. |
| 7. Procedural limitations | Flaws in surgical technique or perioperative care. | - Outdated surgical techniques. - Poor infection control. - Inadequate surgeon experience with anomalies. |

c. *Analytical Themes*

| **Analytical Theme** | **Interpretation** | **Linked Descriptive Themes** |
| --- | --- | --- |
| Systemic inequities | Broader structural issues, including financial, geographical and health system flaws, that delay care and hinder compliance, perpetuating cycles of inequity in LMICs. | 1, 2 |
| Patient and procedural risks | Individual-level factors like anatomy or comorbidities, combined with outdated practices, that elevate complication rates in the absence of adaptive strategies. | 3, 7 |
| Technological and innovation shortfalls | Techniques and devices that lag behind global standards, exacerbating procedural risks. | 4 |
| Capacity constraints | Shortages in skilled workforce and follow-up services that impair delivery of care and monitoring, leading to unmanaged complications. | 5, 6 |

2. **Thematic analysis of facilitators to safe surgery**
   a. *Line-by-line coding*

| **Study** | **Codes** |
| --- | --- |
| Daneshi (2015) | technological advancements in devices, good preoperative planning, staff education, vaccination benefits |
| Dankuc (2015) | use of advanced surgical tools, timely vaccination |
| Yepez-Pabon (2015) | intraoperative diagnostic tools, postoperative imaging, health system financial coverage, compliance with follow-up, long-term follow-up capacity |
| Alcas (2016) | MDT availability, tertiary referral centre, access to advanced imaging, public funding subsidies, access to high-quality devices |
| Chen (2017) | preoperative imaging for risk prediction, surgical planning for anomalies, experienced surgeons for anomalies, intraoperative adjustments, standardised surgical technique |
| Devesahayam (2018) | national policy for standards, standardised surgical technique, avoidance of high-risk techniques, facial nerve monitoring, supervision by senior surgeons |
| Diab (2018) | supervision by senior surgeons, low complication rates via experience, regular follow-up schedule, targeted infection treatment |
| Kasemsiri (2018) | specialist centres for majority of cases, skilled surgeons in specialist centres, follow-up compliance in selection criteria |
| Quang (2019) | tertiary centre access, intraoperative monitoring, skilled surgeons in tertiary setting, good preoperative planning, minimally invasive techniques |
| Lai (2020) | preoperative imaging for risk prediction, surgical planning for anomalies, experienced surgeons for anomalies, intraoperative adjustments, standardised surgical technique |
| Natarajan (2020) | disease clearance before implantation, tertiary ENT centre capabilities, comprehensive preoperative assessments, comprehensive postoperative assessments |
| Haque (2021) | meticulous anaesthetic management, haemodynamic stability for bloodless field, neuromuscular monitoring, device protection during surgery, postoperative symptom control |
| Ahmed (2021) | improved audiological follow-up, improved surgical follow-up, good preoperative planning, soft and safe surgical techniques |
| Huang (2021) | comprehensive preoperative assessment, appropriate surgical approach selection, strict operative indications |
| Piromchai (2021) | tertiary centre access |
| Guo (2022) | N/A |
| Pradhananga (2022) | single experienced surgeon, intraoperative complication management, use of reconstructive materials, clinician education, increased accessibility via collaborations |
| AlKhtoum (2022) | well-trained surgeons, standardised surgical techniques, facial nerve monitoring, perioperative imaging, perioperative antibiotics, compression dressings |
| Castro (2022) | specialised referral centre, basic perioperative care, early postoperative appointment |
| Ozdemir (2022) | tertiary centre, experienced surgeon, standardised surgical technique, facial nerve monitoring, technical modifications over time, perioperative antibiotics, postoperative imaging, complication management strategies |
| Sinha (2022) | diligent preoperative assessment, MDT involvement, surgical technique modifications, perioperative antibiotics, perioperative vaccinations, strict patient selection criteria, state government financial aid |
| Sharma (2022) | tertiary centre, preoperative imaging, MDT assessment, anaesthetic assessment, experienced surgeon, standardised technique, vaccination |
| Jaiswal (2023) | standardised surgical technique, standardised device |
| Mathews (2023) | standardised surgical technique, supervision by senior surgeons, tertiary centre access, intraoperative monitoring, complication management strategies |
| Orlando (2024) | tertiary centre, skilled surgeons for complex cases, high-quality imaging, vaccination for prevention |
| Aqib (2025) | tertiary centre, skilled surgeons, standardized technique, reliable technique |
| Li (2025) | standardised minimally invasive technique, consistent surgical team, preoperative assessments, intraoperative assessments, postoperative assessments, intraoperative steroids |
| Sarkar (2020) | diligent preoperative evaluation, appropriate patient selection |
| Souza (2025) | N/A |
| Zizlavsky (2025) | centralised specialist centres, experienced surgeons |

b. *Descriptive Themes*

| **Descriptive Theme** | **Description** | **Example Codes** |
| --- | --- | --- |
| 1. Surgical expertise and supervision | Enhancing safety with experienced surgeons performing or supervising procedures. | - Supervision by senior surgeons. - Experienced surgeons for anomalies. - Staff education. |
| 2. Standardised and minimally invasive techniques | Using consistent and low trauma approaches to minimise risks. | - Standardised surgical technique. - Avoidance of high-risk techniques. - Minimally invasive techniques. |
| 3. Preoperative planning and intraoperative interventions | Leveraging imaging, assessments and monitoring to mitigate surgical risks. | - Preoperative imaging for risk prediction. - Good preoperative planning. - Intraoperative monitoring. |
| 4. Postoperative care and follow-up | Strategies for monitoring patients postoperatively and ensuring compliance with follow-up. | - Regular follow-up schedule. - Postoperative assessments. - Postoperative symptom control. |
| 5. Specialist centres and infrastructure | Delivering high quality care from referral centres with advanced facilities. | - Tertiary referral centre. - Centralised specialist centres. - Specialised referral centre. |
| 6. Prophylactic interventions | Using prophylactic interventions to avoid complications. | - Timely vaccination. - Vaccination for prevention. - Perioperative antibiotics. |
| 7. Financial and policy support | Improving equity and adherence with funding and policy. | - Public funding subsidies. - State government financial aid. - National policy for standards. |
| 8. Multidisciplinary team collaboration | Collaboration of various healthcare workers | - MDT assessment. - MDT involvement. - Increased accessibility via collaborations. |

c. *Analytical Themes*

| **Analytical Theme** | **Interpretation** | **Linked Descriptive Themes** |
| --- | --- | --- |
| Expertise-driven risk mitigation | Minimising intraoperative risks by using experienced surgeons and standardised techniques. | 1, 2 |
| Widening access to advanced infrastructure and technology | Implementing supportive policies to overcome infrastructural and economic hurdles in LMICs, to enhance access to specialised facilities. | 5, 7 |
| Proactive planning and prevention | Mitigating surgical risk through preoperative planning and prophylactic interventions, as well as intraoperative monitoring. | 3, 6 |
| Holistic team-based continuity | Fostering collaborative and ongoing care to ensure comprehensive patient management from surgery through recovery. | 4, 8 |
